# Antibacterial Thin Films Deposited from Propane–Butane Mixture in Atmospheric Pressure Discharge

**DOI:** 10.3390/ijms24021706

**Published:** 2023-01-15

**Authors:** Pavel Sťahel, Věra Mazánková, Daniela Podzemná, Erika Podzemná, Veronika Pizúrová, Jana Jurmanová, Lubomír Prokeš, Marián Lehocký, Kadir Ozaltin, Hana Pištěková, David Trunec

**Affiliations:** 1 Department of Physical Electronics, Faculty of Science, Masaryk University, Kotlářská 2, 611 37 Brno, Czech Republic; 2 Department of Mathematics and Physics, Faculty of Military Technology, University of Defence, Kounicova 65, 662 10 Brno, Czech Republic; 3 Institute of Physical and Applied Chemistry, Faculty of Chemistry, Brno University of Technology, Purkyňova 118, 612 00 Brno, Czech Republic; 4 Centre of Polymer Systems, Tomas Bata University in Zlín, Trida Tomase Bati 5678, 760 01 Zlín, Czech Republic; 5 Faculty of Technology, Tomas Bata University in Zlín, Vavreckova 275, 760 01 Zlín, Czech Republic

**Keywords:** antibacterial thin films, plasma polymer, propane–butane mixture

## Abstract

Antibacterial coatings on biomedical instruments are of great interest because they can suppress bacterial colonization on these instruments. In this study, antibacterial polymeric thin coatings were deposited on teflon substrates using atmospheric pressure plasma polymerization from a propane–butane mixture. The plasma polymerization was performed by means of surface dielectric barrier discharge burning in nitrogen at atmospheric pressure. The chemical composition of plasma polymerized propane–butane films was studied by energy-dispersive X-ray spectroscopy (EDX) and FTIR. The film surface properties were studied by SEM and by surface energy measurement. The EDX analysis showed that the films consisted of carbon, nitrogen and oxygen from ambient air. The FTIR analysis confirmed, in particular, the presence of alkyl, nitrile, acetylene, imide and amine groups. The deposited films were hydrophilic with a water contact angle in the range of 13–23°. The thin film deposited samples were highly active against both *S. aureus* and *E. coli* strains in general. On the other hand, the films were cytocompatible, reaching more than 80% of the cell viability threshold compared to reference polystyrene tissue.

## 1. Introduction

Antibacterial thin films (coatings) deposited on biomedical devices and implants can remove or reduce bacteria colonization on their surfaces, thus suppressing patient nosocomial infections [1]. Antibacterial coatings can either inhibit the adhesion of bacteria (antifouling coatings) or kill the bacteria on the surface and/or in its vicinity (bactericidal coating) or combine both mechanisms [2]. The recent development of antibacterial coatings include, for example, sharkskin-patterned surfaces [3], nanostructured hierarchical diamond films [4] or silver nanocluster–silica composite antibacterial coatings [5]. Recently, it was found that hydrophilic polymers can provide anti-biofouling properties to surfaces [6]. Several hydrophilic polymers were employed as candidates to develop hydrophilic anti-biofouling coatings. Among them, poly(ethylene glycol) (PEG), zwitterion-containing polymers, and their derivatives, were widely investigated [7]. The most common biopassive coatings currently used are made of PEG and PEG-copolymers [8]. However, the degradation of PEG coatings raises concerns [9]. Poly(2-oxazoline) (POx) thin films are an example of antifouling coatings [10], which could replace PEG coatings. The formation of antibacterial coatings using conventional methods can be a slow and complex multistep procedure. On the other hand, plasma polymerization is known to be a suitable method for the depositing of many biomaterial coatings [11]. Plasma polymerization is the process of creating a highly-branched polymer by plasma-initiated polymerization of the gas precursor. Typically, plasma polymer is created as a thin layer consisting of short chains with random organization and a high degree of crosslinking [12]. So, the plasma polymer is a different material compared with the polymer prepared by conventional chemical polymerization. Plasma polymerization can be performed in low pressure plasma deposition systems or in atmospheric pressure (AP) plasma deposition systems. Thin film depositions at AP allow easy, fast, and continuous processing, due to the application of open systems, without needing an expensive vacuum system. The deposition at AP can have a high deposition rate (several tens of nm per second). The main drawback of AP systems is inadequate uniformity of the deposited films.

In our previous studies, POx thin coatings were deposited on glass substrates using plasma polymerization with 2-methyl-2-oxazoline or 2-ethyl-2-oxazoline vapor as a monomer [13,14]. The plasma polymerization was performed by means of dielectric barrier discharge burning in nitrogen at atmospheric pressure. After tuning the deposition parameters (i.e., monomer flow rate and substrate temperature), stable POx films, which resisted bacterial biofilm formation and had cell-repellent properties, were achieved. However, it was not possible to deposit stable POx films on polytetrafluoroethylene substrates, as the films were soluble in water.

This paper describes plasma polymerization of thin films from a propane–butane (PB) mixture. The plasma polymerization was performed in atmospheric pressure surface dielectric barrier discharge burning in nitrogen with PB admixture. The films were deposited on polytetrafluoroethylene substrates. Polytetrafluoroethylene is one of the fluoroplastics that have excellent thermal, chemical and other properties, but their toxicity still does not meet a satisfactorily adequate level. Toxicity may result from the fluoropolymer itself or the solvent in which it is delivered [15]. Due to this fact, it is of paramount importance to improve non-adequate interaction with materials of biological origin when favorable mechanical properties are intact. The most important strategy would be toxicity minimization and improvement of ability to fight infection by deposition of a thin antibacterial layer. It was already shown that thin films deposited from a propane–butane mixture in nitrogen atmospheric pressure discharge are highly hydrophilic [16]. Now, it was also shown that such thin films also have strong antibacterial properties. This had not been observed and reported till now. So, a new type of antibacterial coating was developed, the deposition of which is easy and cheap. The coating deposition is also fast and it can be done online.

## 2. Results and Discussion

### 2.1. Surface Characterization

**Figure 1:**
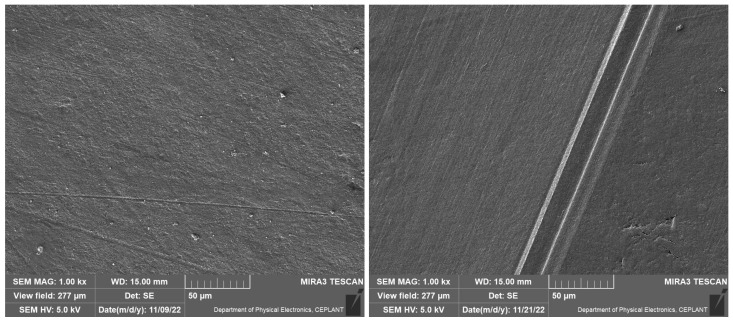
SEM image of PB thin film deposited at the monomer flow rate 35 sccm (**left** image) and SEM image of bare teflon (**right** image).

The surface topography of the studied film is shown in Figure 1. The unevenness of the films, which can be seen in the SEM image in the form of furrows, was caused by the unevenness of the teflon surface. The elemental composition of PB films deposited at different monomer flow rates determined by EDX is shown in Table 1.

The carbon content in the films increased with increasing PB flow rate, and the nitrogen content decreased with increasing PB flow rate. Since the deposition setup was not closed in a vacuum chamber, ambient air could diffuse in the discharge and, therefore, oxygen was also present in the films. The elemental composition of the PB films was close to the elemental composition of POx films [13,14].

The contact angles between the test liquids and PB films were measured using the sessile drop technique in order to determine the total surface free energy and its components. Three test liquids (distilled water, glycerol and diiodomethane (CH${}_{2}$I${}_{2}$)) were used. The acid–base theory, with multiple regression [17], was used to calculate the total surface free energy and its components—the Lifshitz–van der Waals (LW) interaction component and the acid–base (AB) interaction component. The surface free energy and its above-mentioned components of PB films are given in Table 2.

The deposited films were hydrophilic with the water contact angle in the range of $13–23^{{}^{\circ}}$ and the surface free energy in the range of 58–61 mJ/m${}^{2}$.

**Figure 2:**
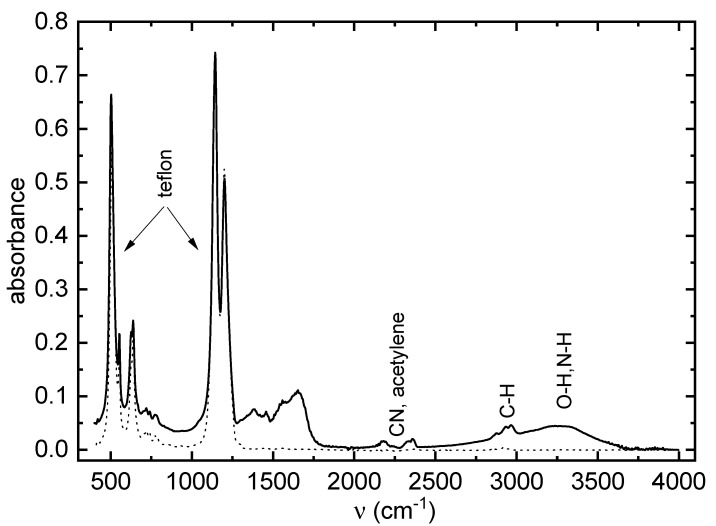
FTIR spectrum of PB thin film deposited at the monomer flow rate of 65 sccm (full line) and FTIR spectrum of bare teflon (dotted line).

The FTIR spectrum of deposited PB thin film is shown in Figure 2.

A wide absorption band between 3500 and 2600 cm${}^{-1}$ indicated the presence of O–H and/or N–H bonds (or hydrogen bonds), related to the hydrophilic properties of the material [18,19]. The 2950 and 2855 cm${}^{-1}$ bands corresponded to C–H bonds in alkyl groups, and they were more intense in samples deposited at flow rates of 65 sccm and 80 sccm, compared to the other samples. In the case of the alkyl absorption band around the wave number of 1400 cm${}^{-1}$, the difference between the samples was insignificant. Absorption bands between 2400 and 2000 cm${}^{-1}$ were characteristic for the presence of triple bonds in the nitrile (CN) and acetylene groups. Absorption bands in the region of 1800–1500 cm${}^{-1}$ were related to the presence of double bonds in the alkene, aromatic, carbonyl, carboxylic, amide and imine groups, and N–H bonds in the amines. Absorption bands at 1200, 1145, 640, 554 and 503 cm${}^{-1}$ were typical for PTFE material.

Previous studies of discharges in nitrogen with hydrocarbons admixture support this analysis of the FTIR spectra. The optical spectra were measured in the same experimental setup [20] and CN and O I spectra were observed. Moreover, the emission line of Hγ and bands of NH and CH were observed in the optical spectra from the discharge in N${}_{2}$/CH${}_{4}$/CO${}_{2}$ mixture [21]. Observation of the CN, CH and NH bands demonstrated the rich chemistry occurring in the discharge. FTIR and GC-MS analyses of gaseous products from the discharges in the nitrogen–methane mixture detected hydrogen cyanide, acetylene and acetonitrile as dominant products. Ethane, ethylene and other nitriles were observed [21,22]. Nitrile groups are common products of the reaction of hydrocarbons (e.g., propane and butane) with nitrogen in discharges, see e.g., [23]. Dehydrogenation of hydrocarbons was also observed for hydrocarbons in discharge with nitrogen [23] or oxygen [24]. The mechanism of the formation of different gaseous products in the discharge could be found in [25,26]. The particles presented in the discharge could then create the film on the substrate.

### 2.2. Antibacterial Properties

The results of the number of viable bacteria per cm${}^{2}$ of sample (CFU/cm${}^{2}$), and the values of antibacterial activity (R) against the growth of *Staphylococcus aureus* CCM 4516 and *Escherichia coli* CCM 4517, are given in Table 3. The tests were performed in triplicate, according to the requirements of the ISO 22196 standard. The calculation of antibacterial activity values was performed again according to ISO 22196: 2011. The samples had a very strong effect against both Gram-negative and Gram-positive test strains, and the values of antibacterial activity (R) were ≥4.8 for *S. aureus* and ≥5.9 for *E. coli*. Such values of antibacterial activity represented strong antibacterial properties, according to ISO 20743: 2014. The only exception was the sample deposited at the flow rate of 80 sccm. Only weak antibacterial activity against *S. aureus* was measured for this sample. The lower antimicrobial activity at this flow rate requires further extensive research.

The strong antibacterial activity might be caused by the presence of amines. Their presence was suggested from FTIR spectroscopy results (see Section 2.1). The significant antibacterial properties of amines have been confirmed in several studies [27,28]. Quaternary ammonium salts penetrate the cell wall of bacteria. Afterwards, reactions with lipids or proteins of the cell membrane occur, resulting in the disorganization of structures and leakage of low molecular weight components [29].

### 2.3. Cytocompatibility Results

**Figure 3:**
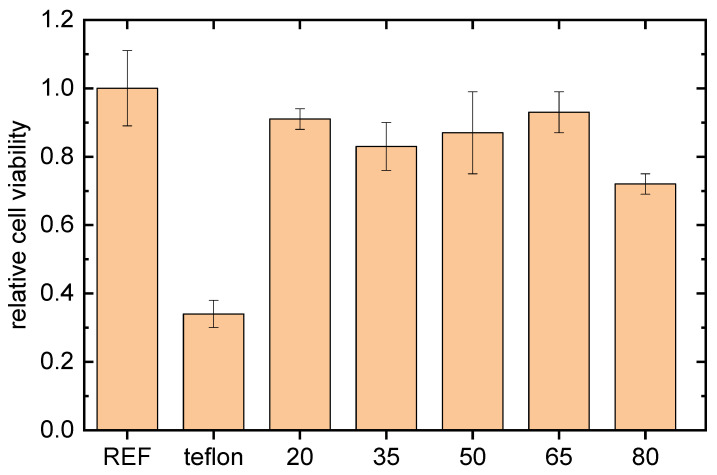
In vitro cytocompatibility results of tested PB films deposited at different flow rates of the monomer. REF marks tissue polystyrene, teflon marks bare substrate and the numbers are monomer flow rates in sccm.

In vitro cytocompatibility results were obtained using mouse embryonic fibroblast (NIH/3T3) cells. The duration of the cell interaction with the tested samples was 48 h. The results obtained are presented in Figure 3, where cell viability for the bare teflon and the film-coated counterparts can be seen, compared to the reference polystyrene tissue. As shown in Figure 3, cell viability on the bare teflon was insufficient, due to the highly hydrophobic nature of the teflon, as seen in Table 2. However, cell adhesion was observed for each coated sample after the coating. Only the sample coated with 80 sccm flow rate exhibited slightly less than 80% cell viability. The highest fibroblast cell viability was observed for the film deposited with 65 sccm flow rate, followed by 20, 50 and 35 sccm, respectively. Each of these was higher than 80% of the cell viability threshold, compared to the reference polystyrene tissue. This result indicated that the film deposition was cytocompatible, besides performing antibacterial properties.

## 3. Materials and Methods

### 3.1. Materials

Polytetrafluoroethylene (PTFE, teflon) foils (TFP universal a.s., Dobřejovice, Czech Republic) with dimensions of 130 mm × 400 mm and thickness 1 mm were used as substrates for deposition. A mixture of propane with butane (84% propane, 15% butane) was used as a monomer for plasma deposition. Antibacterial tests were done with *Staphylococcus aureus* (CCM 4516) and *Escherichia coli* (CCM 4517) both supplied by the Czech Collection of Microorganisms in Brno.

### 3.2. Plasma Deposition

**Figure 4:**
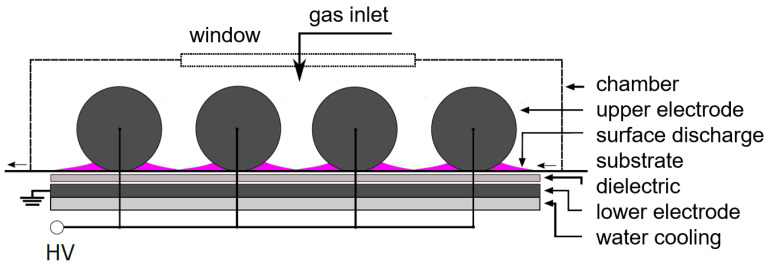
The scheme of deposition setup.

**Figure 5:**
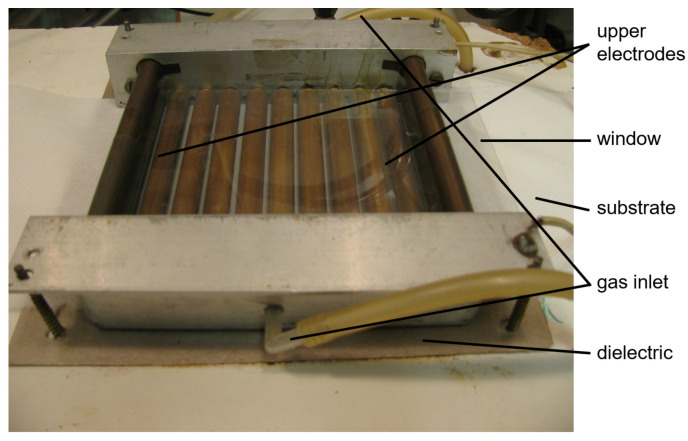
The image of deposition setup.

Plasma polymerization of polymer thin films was performed in a custom built reactor with surface dielectric barrier discharge (SDBD). The electrode system for SDBD consisted of 11 upper revolving cylindrical electrodes made of brass with a length of 10.4 cm and a diameter of 1 cm, between which were gaps with a width of 2 mm. Lower flat electrode with dimensions of 13.6 cm × 10 cm were placed below the cylindrical electrodes and covered by mica dielectrics with 1 mm thickness. The substrate for the deposition was periodically moved between the upper electrodes and lower electrode with a speed of 14.5 cm min${}^{-1}$. The upper electrodes were in contact with the moving substrate and rotated on it. The lower electrode was grounded. The upper electrodes were connected to a high voltage AC power supply (Lifetech, Brno, Czech Republic) using sine wave voltage with an amplitude of 11 kV and frequency of 12 kHz. The input power to the power supply was set to 200 W. The SDBD was then burnt from the upper electrodes across the substrate surface. The schematic drawing and image of the experimental setup are shown in Figure 4 and Figure 5.

Nitrogen, as the working gas, was supplied between the upper electrodes with a flow rate of 6.5 slm. The PB mixture was added to the nitrogen flow with flow rates from 20 sccm to 80 sccm, so the PB concentration was 0.31–1.23%. The deposition setup was placed in a fume cupboard. The substrate was moved through the discharge 3 times, so the deposition time was 6.5 s.

### 3.3. Surface Characterization

The deposited films were imaged with a scanning electron microscope (SEM) MIRA3 (TESCAN, Brno, Czech Republic) with a Schottky field emission electron gun, equipped with secondary electron and back-scattered electron detectors as well as with a characteristic X-ray detector (EDX) analyzer (Oxford Instruments, High Wycombe, UK), which was used to determine the film elemental composition. The IR spectra of deposited films were measured by a FTIR spectrometer Alpha (Bruker, Billerica, MA, USA), using a single reflection ATR module Platinum. The total surface free energy of the films was determined by means of measurements of the contact angles between the testing liquids and the film surfaces using a sessile drop technique. The acid–base theory was used for the calculation of the total surface free energy.

### 3.4. Antibacterial Tests

Before antibacterial testing, samples were disinfected by means of UV radiation, and the polypropylene foil, used in the tests, was disinfected by rinsing with 70% denatured ethanol. Two bacterial strains, gram-negative *Escherichia coli* (CCM 4517) and gram-positive *Staphylococcus aureus* (CCM 4516), were used for the antibacterial tests. The antibacterial testing was performed according to ISO 22196, with modifications. Bacterial suspensions (*Escherichia coli* $2.7\times 10^{5}$ CFU mL${}^{-1}$; *Staphylococcus aureus* $5.4\times 10^{4}$ CFU mL${}^{-1}$) were prepared in 1/500 Nutrient broth (HiMedia laboratories, Mumbai, India ). The bacterial suspension was dispensed on the sample surface (dimensions 25 mm × 25 mm) in the volume 100 μL and the sample was covered with the polypropylene foil (dimensions 20 mm × 20 mm). Samples with foils were cultivated at 35 °C and 100% relative humidity for 24 h. After the incubation time, the polypropylene foil was removed and each sample was completely washed with SCDLP broth (HiMedia laboratories, Mumbai, India), which was subsequently collected. The viable bacterial count was determined by the pour plate culture method (PCA, HiMedia laboratories, Mumbai, India).

### 3.5. Cytocompatibility Test

The mouse embryonic fibroblast continuous cell line (NIH/3T3, ATCC^®^ CRL-1658™, Teddington, UK) was used for the cytocompatibility test, according to the EN ISO 10993-5 standard, with modification. As a culture medium, the ATCC-formulated Dulbecco’s Modified Eagle’s Medium (BioSera, Nuaille, France), containing 10% calf serum (BioSera, Nuaille, France) and Penicillin/Streptomycin at 100 U mL${}^{-1}$ (PAA Laboratories GmbH, Pasching, Austria) was used. The tested samples were prepared with a dimension of 10 mm × 10 mm and sterilized by UV radiation (wavelength of 258 nm) for 30 min and placed into the 24 well-plate. The cells were seeded onto the samples in the concentration of $1\times 10^{4}$ for an hour for adhesion of the cells. Tissue polystyrene was used as a reference. After the pre-cultivation, a sufficient amount of the medium was added and incubated for 48 h at 37 ${}^{{}^{\circ}}$C. The changes in cell morphology were observed with an inverted fluorescent microscope (Olympus, IX 81; Hamburg, Germany). To assess the cytotoxic effect, an MTT assay (Duchefa, Biochemie, Haarlem, The Netherlands) was performed. The absorbance was measured by an Infinite M200 Pro NanoQuant absorbance reader (Tecan, Männedorf, Switzerland). All tests were performed in triplicate.

## 4. Conclusions

A fast and cheap method for the deposition of antibacterial coatings on teflon surfaces was developed. The deposition time was only 6.5 s, so the deposition could be done online. The coatings deposited at all the used monomer flows were hydrophilic. The coatings deposited with a PB flow rate of up to 65 sccm had a very strong antibacterial activity against *S. aureus* and *E. coli*. This antibacterial activity was even better than the antibacterial activity of POx films [13,14]. This could be due to fact that the hydrophilic poly(2-oxazoline) fragment helped balance the toxicity of antimicrobial quaternary ammonium groups [30]. The PB coatings deposited on glass substrates were also tested for antibacterial activity against *Staphylococcus epidermidis* (CCM 4418) and it was found that the PB films deposited at PB concentrations up to 1% were also active against this bacterial strain [31]. However, with increasing PB concentration the antibacterial activity decreased and the PB films deposited at a PB concentration of 1.9% even promoted cell viability. This was not observed in our experiments, but the PB films were cytocompatible reaching more than 80% of the cell viability threshold, compared to the reference polystyrene tissue.

The antibacterial activity of PB films could be caused by their hydrophilicity and the presence of amines. Due to their having similar elemental composition to that of POx films it could be concluded that the PB films could also have antibiofouling properties.

## Figures and Tables

**Table 1:** The elemental composition of films deposited at different monomer flow rates. The elemental composition is given in atomic %.

Flow Rate (sccm)	C	N	O
20	31.1	48.9	20.0
35	40.2	43.0	16.8
50	47.9	37.5	14.6
65	55.2	31.8	13.0
80	54.4	33.0	12.6

**Table 2:** The contact angles for different liquids and surface free energy, and its components, of PB films deposited at different monomer flow rates. Teflon marks bare substrate.

Flow Rate (sccm)	Contact Angle (${}^{{}^{\circ}}$)			Surface Free Energy (mJ/m${}^{2}$)		
	**Water**	**CH${}_{2}$I${}_{2}$**	**Glycerol**	**Total**	**LW**	**AB**
teflon	83.5 ± 1.2	63.2 ± 1.3	74.7 ± 1.3	30.3 ± 1.0	26.7 ± 0.8	3.5 ± 0.8
20	12.7 ± 1.6	32.8 ± 1.7	58.1 ± 1.7	61.2 ± 3.6	43.0 ± 1.2	18.2 ± 2.5
35	16.2 ± 1.2	37.8 ± 0.8	60.0 ± 4.0	58.8 ± 4.9	40.7 ± 0.4	18.2 ± 4.5
50	21.0 ± 1.3	38.2 ± 1.0	60.9 ± 3.0	58.6 ± 5.4	40.2 ± 0.6	18.7 ± 4.8
65	20.9 ± 1.5	29.4 ± 5.0	62.8 ± 1.0	61.0 ± 2.2	40.1 ± 0.4	20.8 ± 1.8
80	22.8 ± 1.2	38.3 ± 1.1	62.9 ± 3.6	60.8 ± 3.9	40.5 ± 0.5	20.4 ± 3.4

**Table 3:** Resulting numbers of surviving colonies (CFU/cm${}^{2}$) on the PB films deposited at different monomer flow rates. Teflon marks bare substrate.

Flow Rate (sccm)	*S. aureus* CCM 2022 (CFU/cm${}^{2}$)	*E. coli* CCM 4517 (CFU/cm${}^{2}$)
teflon	$6.2\times 10^{4}$	$9.1\times 10^{5}$
20	<1	<1
35	<1	<1
50	<1	<1
65	<1	<1
80	$4.5\times 10^{3}$	<1

## Data Availability

The data presented in this study are available upon reasonable request from the corresponding author.
